# Vestibular Modulation of Sympathetic Nerve Activity to Muscle and Skin in Humans

**DOI:** 10.3389/fneur.2017.00334

**Published:** 2017-07-26

**Authors:** Elie Hammam, Vaughan G. Macefield

**Affiliations:** ^1^School of Medicine, Western Sydney University, Sydney, NSW, Australia; ^2^Neuroscience Research Australia, Sydney, NSW, Australia

**Keywords:** galvanic vestibular stimulation, linear acceleration, muscle sympathetic nerve activity, skin sympathetic nerve activity, vestibular system, vestibulosympathetic reflexes

## Abstract

We review the existence of vestibulosympathetic reflexes in humans. While several methods to activate the human vestibular apparatus have been used, galvanic vestibular stimulation (GVS) is a means of selectively modulating vestibular afferent activity *via* electrodes over the mastoid processes, causing robust vestibular illusions of side-to-side movement. Sinusoidal GVS (sGVS) causes partial entrainment of sympathetic outflow to muscle and skin. Modulation of muscle sympathetic nerve activity (MSNA) from vestibular inputs competes with baroreceptor inputs, with stronger temporal coupling to the vestibular stimulus being observed at frequencies remote from the cardiac frequency; “super entrainment” was observed in some individuals. Low-frequency (<0.2 Hz) sGVS revealed two peaks of modulation per cycle, with bilateral recordings of MSNA or skin sympathetic nerve activity, providing evidence of lateralization of sympathetic outflow during vestibular stimulation. However, it should be noted that GVS influences the firing of afferents from the entire vestibular apparatus, including the semicircular canals. To identify the specific source of vestibular input responsible for the generation of vestibulosympathetic reflexes, we used low-frequency (<0.2 Hz) sinusoidal linear acceleration of seated or supine subjects to, respectively, target the utricular or saccular components of the otoliths. While others had discounted the semicircular canals, we showed that the contributions of the utricle and saccule to the vestibular modulation of MSNA are very similar. Moreover, that modulation of MSNA occurs at accelerations well below levels at which subjects are able to perceive any motion indicates that, like vestibulospinal control of posture, the vestibular system contributes to the control of blood pressure through potent reflexes in humans.

## Overview of Blood Pressure and the Nervous System

The autonomic nervous system controls most visceral functions of the body automatically, without the requirement for conscious control. The efferent outflow comprises three subdivisions: the enteric nervous system, which deals exclusively with gastrointestinal function, and the sympathetic (thoracolumbar) and parasympathetic (craniosacral) nervous systems. The latter two subdivisions are regularly thought of as operating in parallel and antagonistic (1). While this is true in many organs that receive dual innervation, such as the pupil of the eye and the heart, control of blood flow in the systemic circulation is governed exclusively by the sympathetic nervous system (2). Similarly, vestibular-mediated changes in peripheral blood flow are brought about *via* the sympathetic nervous system, giving rise to the term “vestibulosympathetic reflex” (3). Accordingly, this review is focused on the relationship of the vestibular input and its effect on the sympathetic output to muscle and skin. Microelectrode recordings from postganglionic sympathetic axons in motor fascicles of human peripheral nerves have revealed that muscle sympathetic nerve activity (MSNA), which occurs as bursts of activity coupled to the cardiac cycle *via* the arterial baroreflex, consists only of vasoconstrictor impulses (4). Given that the skeletal muscle vascular beds make up a significant proportion of cardiac output, muscle vasoconstrictor drive contributes importantly to the control of blood pressure (5). Conversely, skin sympathetic nerve activity (SSNA) is primarily involved in thermoregulation and emotional expression, supplying cutaneous blood vessels, sweat glands, and hairs (4).

## Vestibulosympathetic Reflexes in Humans

In this review, we explore the role of the vestibular system in cardiovascular control in humans, with particular reference to interactions between the vestibular system and sympathetic outflow to muscle and skin. We shall assume that the reader has a good knowledge of the anatomy and physiology of the vestibular apparatus, and refer the reader to a recent comprehensive review of the anatomical and physiological substrates supporting the existence of vestibulosympathetic reflexes (3). While studies in animals clearly provided overwhelming evidence for the anatomical and physiological pathways describing the influence of the vestibular apparatus on the cardiovascular system, its role in humans has been more difficult to explore and establish. Much like the animal work using different methodologies, research conducted on human participants has employed both physiological and electrical stimulation, with the inherent strengths and weaknesses associated with these approaches. Throughout this review, emphasis will be placed on studies that have directly recorded MSNA and SSNA in awake human subjects *via* intraneural microelectrodes (microneurography). Moreover, we shall aim to highlight evidence in which differences in vestibular modulation of MSNA and SSNA exist.

## Caloric Stimulation

Caloric stimulation is a technique that delivers cold or warm water to the tympanic membrane *via* the ear canal, producing nystagmus (involuntary eye movements) and, hence, indicating that vestibulo–ocular reflexes have been activated. In short, this method produces a thermal gradient within the semicircular canals (the horizontal canals in particular) that leads to increased endolymphatic flow and stimulation of vestibular hair cells. Employing this natural stimulation, Costa et al. (6) recorded MSNA to unilateral caloric stimulation using warm water irrigation but found no evidence of increased sympathetic outflow to the leg (6). On the other hand, Cui and colleagues employed bilateral caloric stimulation, using both hot- and cold-water irrigation, and concluded that caloric stimulation decreases SSNA (7) and transiently increases MSNA and that these responses are proportional to the degree of nystagmus (8). It is not clear why there is a discrepancy in the findings of these two groups, but it is possible that the differences are due to the different means by which caloric stimuli were delivered (9). It is also worth pointing out that Ray and colleagues (10, 11) found no modulation of sympathetic nerve activity to either muscle or skin during active horizontal rotations of the head, another method that stimulates the horizontal semicircular canals. Finally, it is important to note that the aforementioned studies limited the stimulations to the horizontal canals, as the vertical canals cannot be selectively stimulated in human subjects. Of course, while this means that there is a possibility that the vertical canals play a role in cardiovascular control, studies in animals strongly argue against this (12).

## Head-Down Neck Flexion (HDNF)

Another means of stimulating the vestibular apparatus physiologically in humans is HDNF. The method entails laying the subject prone with the head and body aligned and by tilting the head downward the maneuver creates an altered gravitational input to the otolith organs. As a response to this stimulus, Essandoh, Normand, and their colleagues demonstrated decreases in arterial pressure and blood flow to the limbs (13, 14). However, it was Shortt and Ray (15) who recorded MSNA and demonstrated that the method leads to an increase in burst frequency and heart rate—increases that were sustained throughout the 10 min of HDNF (15). The same response was not evident during recording of SSNA (16), emphasizing the independence of sympathetic outflow to muscle and skin. Furthermore, studies outlined that the MSNA response is dependent on the magnitude of the stimulus (17) and is the same to the upper limbs as it is to the lower limbs (18, 19), contrary to what is reported in animal studies (20–22). However, in addition to stimulating both the utricle and saccule, HDNF activates several non-vestibular inputs capable of increasing sympathetic outflow—in particular, afferents from muscle (and other) receptors in the neck (23).

## Off-Vertical Axis Rotation (OVAR)

Another approach to investigate the roles of the otolith organs is *OVAR*—a method well-known to produce motion sickness (24) and widely used to study vestibular–ocular reflexes (25–27). The technique involves continuous horizontal rotation of the seated body at a constant velocity, with—as its name suggests—the axis of rotation being tilted (15°) from vertical. Initially, the semicircular canals are activated by the angular acceleration, but as the fluid in the semicircular canals starts to move at the same velocity as the head, the semicircular canals no longer provide a signal of rotational motion after ~12 s (28). This rotational steady state provides linear acceleration allowing the otolith organs to be activated; by keeping the neck aligned with the body axis, this eliminates neck movement and, in turn, sustaining constant afferent input. Indeed, Yates and Bronstein (29) showed that individual vestibular afferents start to fire as soon as the nose-up position is passed during OVAR (29). In humans, Kaufmann and colleagues applied OVAR while recording MSNA to study the vestibulosympathetic reflex across a wide range of rotational velocities and found that in the nose-up position there was an increase in MSNA to the lower limbs. The reflexive increase in neural traffic occurred within a short latency (0.4 s)—too quick to be attributed to the baroreflex that has a minimal latency of 1.22 s to the lower limbs (30). These data provided evidence of a vestibulosympathetic reflex, originating from the otolith, contributing to peripheral blood pressure control. Indeed, a recent study that applied centrifugal forces to astronauts pre and post spaceflight demonstrated that depression of otolithic function following space travel leads to a temporary depression of blood pressure control on Earth—a transient dysfunction that reverses with the “re-conditioning” of the gravitational accelerometers, i.e., the otolithic organs (31). By contrast, OVAR applied in the nose-down position caused a decrease in MSNA (32)—results conflicting those seen in HDNF (15). The discrepancies shown in these studies may be due to the different means of stimulating the vestibular apparatus, such as changes in posture and, hence, changes in vestibular input with respect to gravity, and the use of dynamic stimuli and neck displacements in one study but not in the other study. Accordingly, this leads to a need for an experimental design to selectively activate vestibular inputs without acting on other non-vestibular inputs.

## Galvanic Vestibular Stimulation (GVS)

Galvanic vestibular stimulation is a means of stimulating afferents in the vestibular nerves through weak electrical stimuli applied to the mastoid processes, and was initially used to study the contributions of the vestibular system to control of eye movements, locomotion, and posture (33, 34) and has since been used by our group to study vestibulosympathetic reflexes in humans. It provides a selective form of stimulation to the vestibular apparatus (35–37), though it has been pointed out that GVS is less selective than other methods, resulting in an overall stimulation of vestibular nerves rather than specific components (9). Goldberg et al. (35) made direct recordings from vestibular afferents in primates during application of GVS. They showed in the squirrel monkey that when cathodal GVS was applied in the perilymphatic space, or anodal GVS applied at a more proximal point, both caused excitatory responses in vestibular afferents (35). GVS stimulates the hair cell axon terminals of the vestibular afferents and alter their firing (35). Cathodal currents depolarize and, thus, increase the firing rate of vestibular afferents, whereas anodal currents hyperpolarize and thereby decrease their firing rate. As noted above, a limitation of GVS is that it cannot discriminate between the vestibular end organs (semicircular canals or otolith organs). However, animal research has shown that the response to GVS is predominantly otolithic (38–40), and other evidence strongly argues against a contribution from the semicircular canals in the vestibular control of sympathetic nerve activity (6, 10, 11). Thus, one can assume that any changes in sympathetic outflow in humans during GVS can be attributed to activation of the otolith organs (9, 38–40). A particular advantage offered by GVS is that it is selective to the vestibular system: it does not modulate neck afferents (as in *HDNF*), cause fluid shifts in the body (as in *OVAR*), or influence any other physiological parameter that may affect sympathetic outflow—such as heart rate, blood pressure, or respiration (9, 33).

Bolton et al. (41) first applied GVS in the form of a 1 s step to examine the vestibular contributions to cardiovascular control, in particular its effect on sympathetic outflow to muscle vascular beds in the lower limbs. The investigators found that the application of a 2 mA current across the mastoid processes in a binaural, bipolar fashion adequately modified the firing of vestibular afferents because subjects reported strong perceptual illusions of sway toward the anode. However, despite being delivered at different times following the heart beat, with a delay of 0, 200, 400, or 600 ms following the R-wave of the ECG, GVS failed to cause a net change in MSNA but did cause short-latency bursts of SSNA. Therefore, it was concluded that the short duration of electrical vestibular stimuli did not interact with the baroreceptors, nor did they cause modulation of MSNA, but did excite cutaneous vasoconstrictor and sudomotor neurones (41).

**Figure 1:**
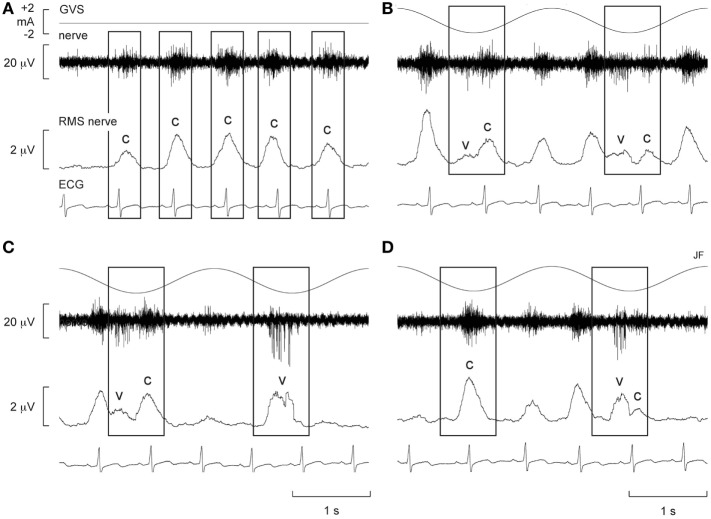
Spontaneous muscle sympathetic nerve activity (MSNA) presented as the filtered neurogram (nerve) and as an RMS-processed signal (RMS nerve), shown with ECG at rest **(A)** and during sinusoidal GVS (sGVS) at 0.5 Hz **(B–D)**. Each panel spans a 4 s period. **(B–D)** Consecutive sequences obtained during sGVS at 0.5 Hz to illustrate the coupling of MSNA to the ECG and to the vestibular input. The rectangles illustrate the relationship between the sympathetic burst and the cardiac rhythm (c) and the vestibular rhythm (v). Reproduced with permission from Bent et al. (46).

Alternatively, Voustianiouk et al. (42) employed dynamic stimuli in the form of brief trains (30 ms) of 10 pulses of GVS and found a clear modulation of MSNA. While many animal studies demonstrated cardiovascular responses to trains of electrical stimuli delivered to the vestibular system (43–45), Voustianiouk and colleagues clearly showed that short-latency vestibulosympathetic reflexes do exist in humans. The authors concluded that these reflexes might contribute to the control of arterial blood pressure, especially during rapid postural changes (42). To further investigate, our laboratory used continuous (as opposed to intermittent) dynamic GVS to study vestibular modulation of muscle sympathetic outflow (46). Vestibular afferents were stimulated using continuous sinusoidal (0.5–0.8 Hz) stimulation (2 mA) to the mastoid processes (46). Participants experienced strong perceptual illusions of “rocking in a boat” or “swinging from side to side in a hammock,” at a frequency matching that of the stimulation. Interestingly, this study showed that overall MSNA increased by 156% and that sinusoidal GVS (sGVS) was able to cyclically modulate MSNA (Figure 1). For this and subsequent studies using sGVS, we used cross-correlation analyses to identify temporal coupling between the occurrence of sympathetic nerve activity and the peak of the sinusoidal vestibular stimulus (i.e., the peak of the sinusoid) or the peak of the ECG (i.e., the R-wave). This approach allows one to quantify vestibular or cardiac modulation in terms of the modulation index—the magnitude of the temporal coupling of sympathetic outflow to a reference event, be it the vestibular stimulus or the heart beat.

**Figure 2:**
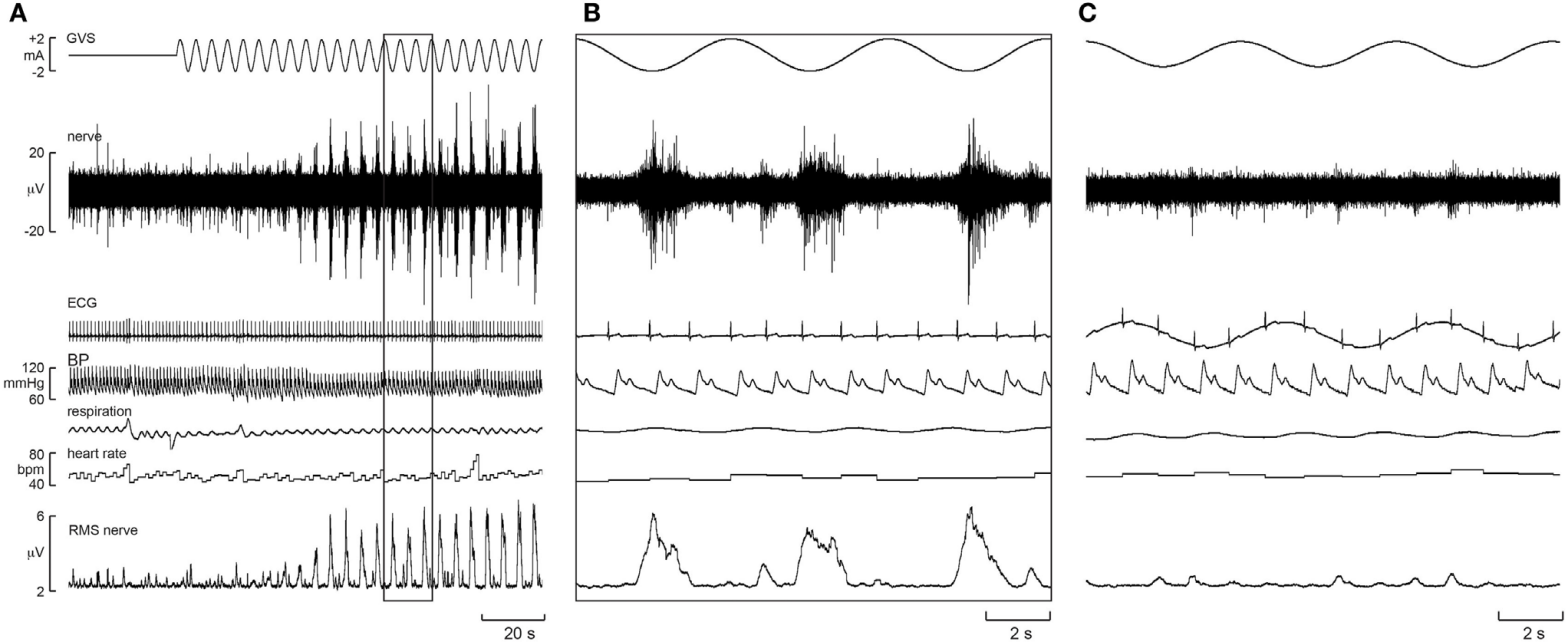
Experimental records of muscle sympathetic nerve activity (MSNA) from one subject during sinusoidal GVS (sGVS) at 0.8 Hz, showing super entrainment of MSNA to the sinusoidal vestibular input. The highlighted section in panel **(A)** is shown expanded in panel **(B)**. **(C)** Delivery of sGVS to the shoulders (anode on right shoulder, cathode on left). Reproduced with permission from Macefield and James (47).

Furthermore, there was evidence of generation of *de novo* sympathetic bursts to the dynamic GVS: two bursts of MSNA could be generated per cardiac interval (46), with one burst being temporally coupled to the sinusoidal vestibular input and the other to the baroreceptor (cardiac) input (Figure 1). This observation showed that the vestibular system exerts a significant influence on sympathetic outflow to muscle, and that this may operate independently of the arterial baroreceptors in the control blood pressure (46). Recently, we provided evidence of “super entrainment” of MSNA, in which a burst of MSNA is very strongly coupled to a phase of sGVS (47). As shown in Figure 2, this strong temporal coupling was clearly generated by stimulation of the vestibular nerves, as applying the same current to the shoulders failed to induce any modulation.

**Figure 3:**
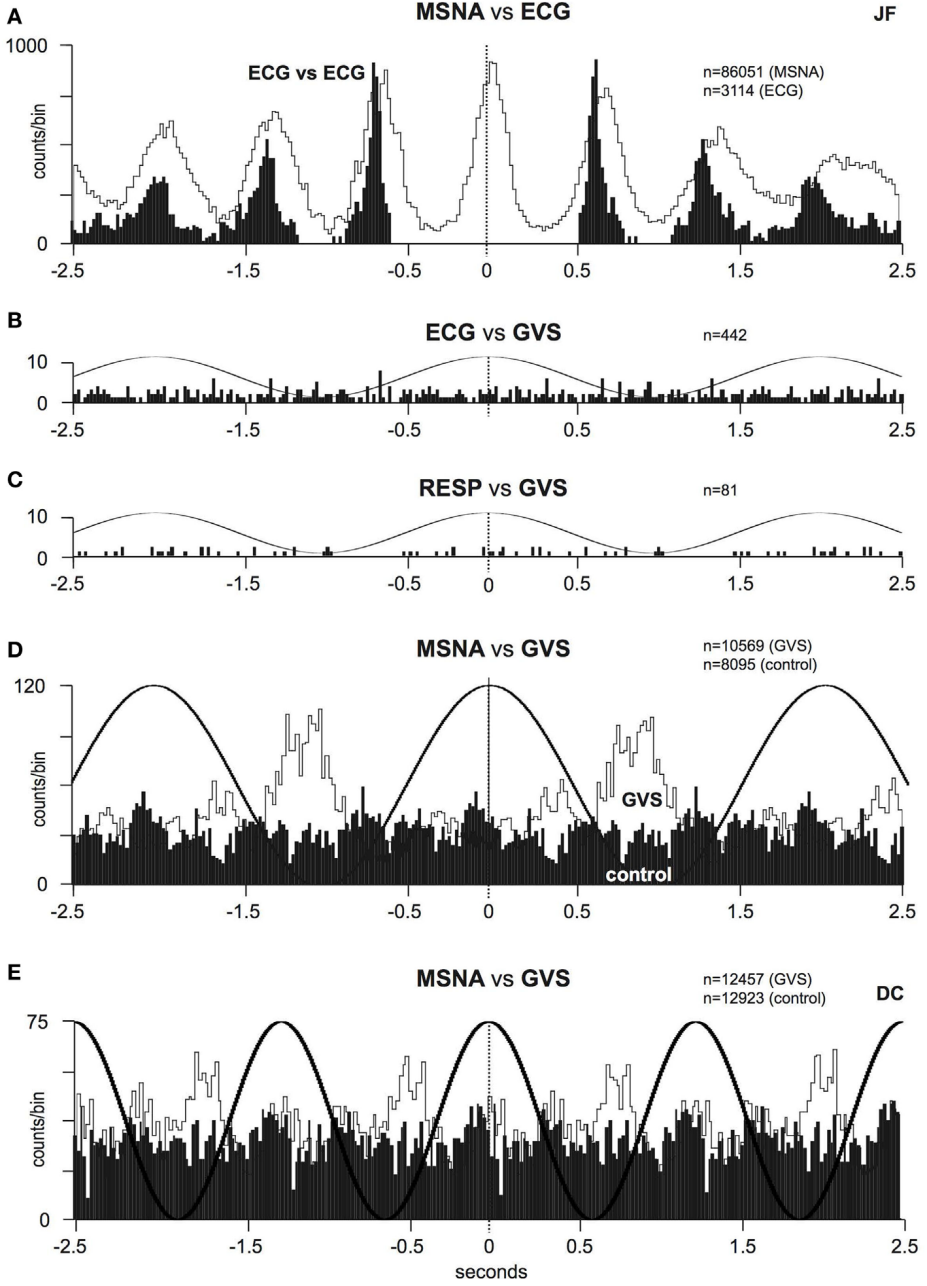
**(A)** Cross-correlation histograms of the relationship between muscle sympathetic nerve activity (MSNA) and R-waves of the ECG (white histogram) and autocorrelogram of the ECG (black histogram). **(B,C)** Cross-correlation histograms between ECG and sinusoidal GVS (sGVS) and respiration (inspiratory peaks) and sGVS. A 0.5 Hz sine wave has been superimposed on the histogram to illustrate the timing of the galvanic vestibular stimulation; it has been inverted for clarity. **(D,E)** Cross-correlation histograms of MSNA with respect to the vestibular input (GVS), in white, or to a control sine wave (control), in black. Data in panels **(A–D)** are from the same subject represented in Figure 1; data in panel **(E)** were obtained from another subject. 20 ms bins in all panels. *n* = the numbers of counts comprising the histograms. Reproduced with permission from Bent et al. (46).

To further explore the effect of sGVS on sympathetic outflow, in separate studies, our laboratories exposed participants to a wider range of frequencies (0.2–2.0 Hz, 200 cycles, ±2 mA) during recordings of both muscle and SSNA (48, 49). Similarly, in both of these studies, all of the subjects reported robust vestibular illusions, though these were reduced at the higher frequencies. Cross-correlation analysis revealed partial phase locking of both muscle and skin to the cyclic vestibular input, with the vestibular modulation of MSNA found to be greatest at 0.2 Hz and lowest at 0.8 Hz—the latter being the frequency closest to the cardiac rhythm. Figure 3 shows cross-correlation histograms between MSNA and ECG (Figure 3A) and 0.5 Hz sinusoidal GVS (Figure 3D). It can be seen that the cardiac modulation of MSNA is higher than the vestibular modulation, and that GVS has no direct effect on ECG (Figure 3B) or respiration (Figure 3C). The cross-correlation histogram between MSNA and GVS, delivered at 0.8 Hz, is shown for another subject in Figure 3E. Unlike the vestibular modulation of MSNA, the vestibular modulation of SSNA was high at all frequencies of stimulation. This prompted further investigation to better understand the modulation of MSNA. In an extension of the latter study, sGVS was delivered at the resting heart rate of a given subject and at frequencies (0.1, 0.2, 0.3, and 0.6 Hz) above and below the central cardiac frequency. Results confirmed that vestibular modulation of MSNA was significantly reduced when it coincides with the cardiac rhythm, confirming the competitive nature of vestibular and baroreceptor inputs. This further highlights the dominance of the arterial baroreceptors in modulating MSNA (50).

**Figure 4:**
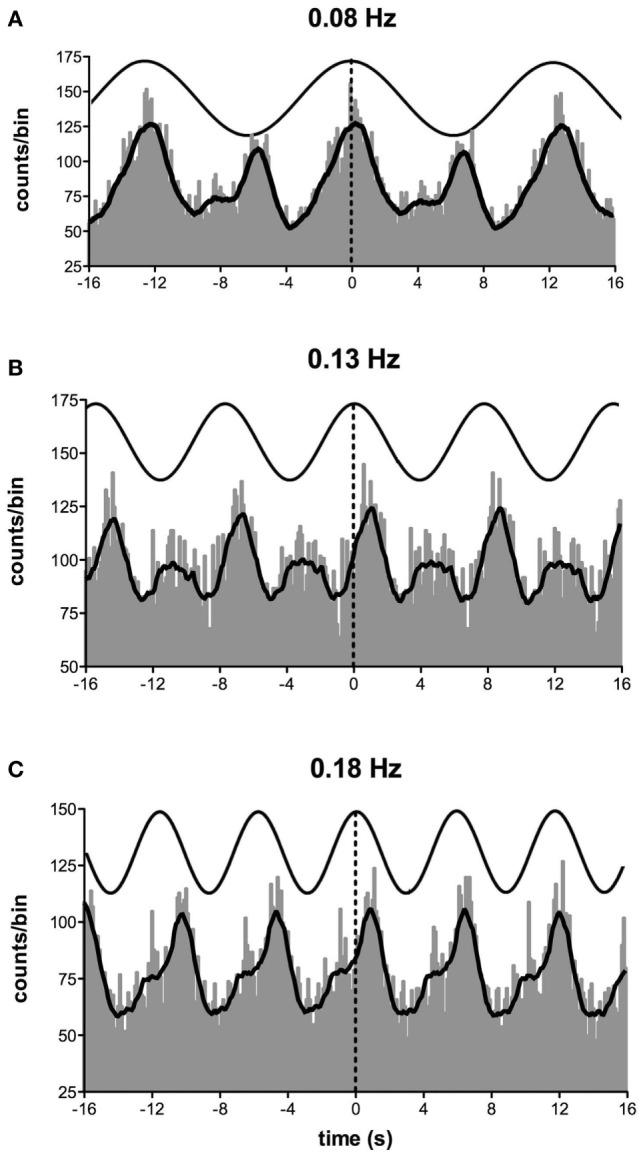
Cross-correlation histograms between muscle sympathetic nerve activity and sinusoidal GVS in one subject. The thick curve superimposed on the histograms is the smoothed polynomial that was fitted to the data. The sinusoid above represents the galvanic stimulus, delivered at **(A)** 0.08, **(B)** 0.13, and **(C)** 0.18 Hz. Each cross-correlation histogram shows a large peak of modulation (primary peak), associated with the positive peak of the sinusoid, and a smaller peak (secondary peak). The secondary peak was largest at 0.08 Hz and smallest at 0.18 Hz. Reproduced with permission from Hammam et al. (51).

Furthermore, as the highest modulation of sympathetic outflow in the study by Grewal et al. (48) occurred at 0.2 Hz, we explored whether lower frequencies of stimulation (0.08–0.18 Hz) produce higher or lower modulation. These are frequencies specifically associated with very slow postural displacements (i.e., such as those experienced during tall building sway, evoking motion sickness) (51, 52). Analysis of the sympathetic discharge revealed that such low-frequency GVS induces two peaks of modulation of MSNA per cycle of stimulation—one associated with the positive peak and the other with the negative peak (Figure 4) (51). This observation also held true when recording sympathetic activity to the skin (52).

**Figure 5:**
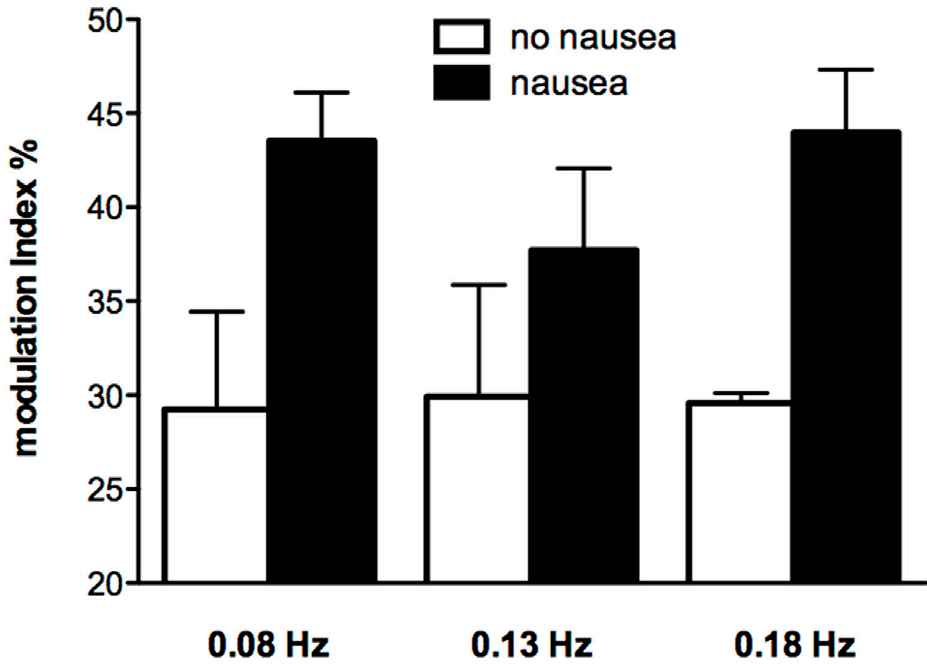
Modulation indices of primary peak of skin sympathetic nerve activity during sinusoidal GVS at different frequencies as a function of whether or not subjects reported nausea. It can be seen that modulation indices were higher in those subjects who reported nausea. Reproduced with permission from Hammam et al. (52).

While very few subjects reported nausea at the high frequencies of sGVS, half of the subjects experienced nausea at frequencies of sGVS < 0.2 Hz. Moreover, those subjects who did report nausea displayed a greater vestibular modulation of SSNA, as shown in Figure 5 (52). It is interesting that this augmented vestibular modulation of sympathetic outflow to skin was not generalized to the sympathetic outflow to muscle (53). However, perhaps this is not surprising, given that increases in SSNA explain two of the features of nausea—pallor and sweating.

In order to explain the two-peak response, it is noteworthy that in all experiments we recorded sympathetic nerve activity from the left side (51, 52). In addition, the anode was always located over the right mastoid process, making it more straightforward in interpreting the results. The larger (primary) peak (see Figure 4A) was related to the positive phase of the sinusoid (i.e., 0 to 2 mA), while the smaller (secondary) peak was related to the negative phase. Given that hyperpolarization of the vestibular nerves occurs at the anode and depolarization at the cathode (37), we can see that the positive phase of stimulation corresponds to hyperpolarization on the right side. Naturally, because we are applying current bilaterally (across both mastoid processes) hyperpolarization on the right side means that the left side is being depolarized. As the current slowly shifts toward the left side, it causes hyperpolarization on this side but depolarization on the right. We suggest that this secondary depolarization is responsible for the secondary burst of modulation. This pattern of modulation—a primary peak corresponding to the positive phase of sGVS and a smaller secondary peak corresponding to the negative phase—matched the stimulus frequency but was never observed at the higher frequencies of stimulation used previously (46, 48, 49), presumably because at frequencies >0.2 Hz there is not enough time for a second peak to be seen.

**Figure 6:**
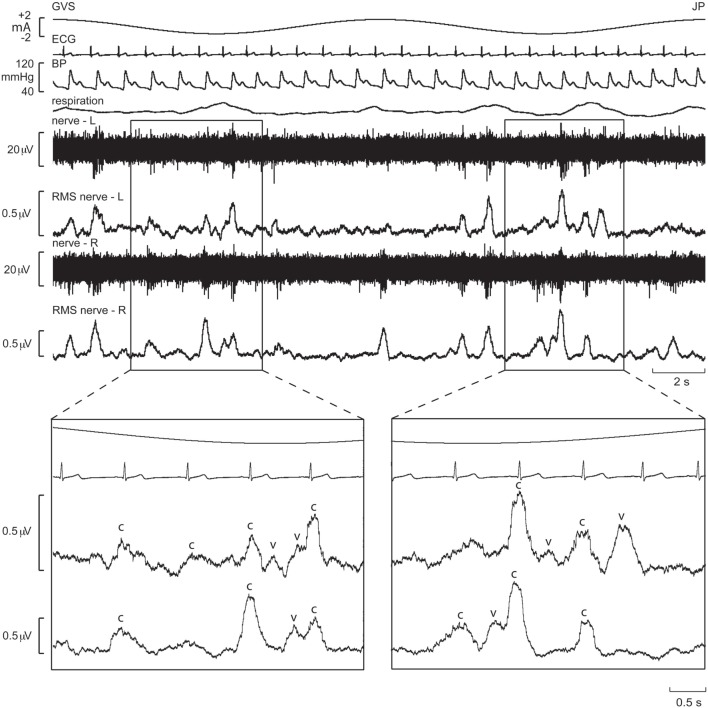
Bilateral recordings of muscle sympathetic nerve activity, together with ECG, blood pressure, and respiration, during sinusoidal GVS (galvanic vestibular stimulation) at 0.08 Hz in one subject. Overall, sympathetic outflow was similar between the two sides, but close inspection revealed subtle differences. In the expanded sections, the sympathetic bursts have been shifted back 1.25 s in time to account for peripheral conduction delays, allowing those bursts aligned with the cardiac cycle (“c”) or vestibular stimulus (“v”) to be identified. Reproduced with permission from El Sayed et al. (58).

**Figure 7:**
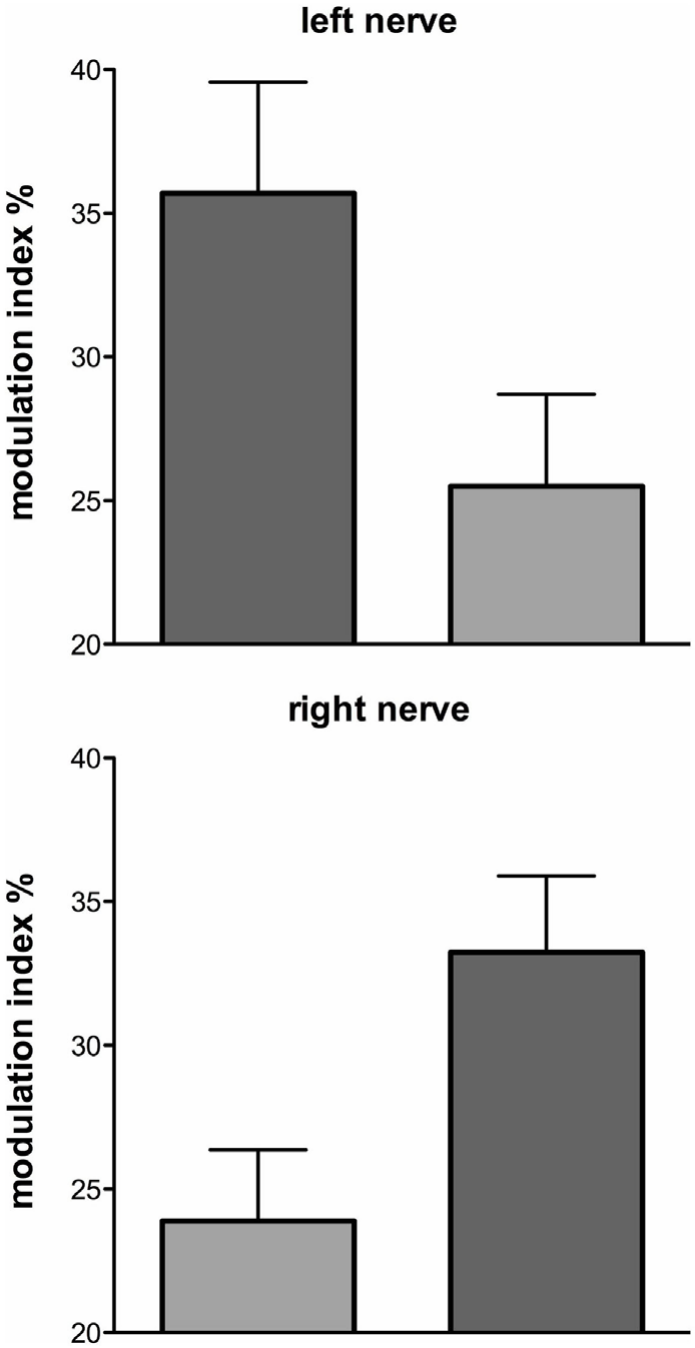
Mean ± SE modulation indices for the primary (dark gray) and secondary (light gray) peaks of modulation of muscle sympathetic nerve activity. Data obtained from 10 subjects. Reproduced with permission from El Sayed et al. (58).

These series of studies showed that cyclically changing vestibular nerve input could generate a marked modulation of muscle vasoconstrictor activity (46, 48, 49, 51). This most likely acts through the rostral ventrolateral medulla (RVLM), which is the primary output nucleus for muscle vasoconstrictor neurones (54, 55) and receives direct excitatory inputs from the otoliths (56, 57). So, the frequency-dependant modulation of MSNA may reflect vestibular inputs arriving from both sides projecting onto RVLM. This was later confirmed during experiments involving bilateral recordings of MSNA, where cross-correlation analysis did indeed reveal a reversal of modulation in the primary and secondary peaks recorded from the left and right sides: a primary peak on the left was associated with a secondary peak on the right and a secondary peak on the left was associated with a primary peak on the right (Figures 6 and 7). This is probably of greater interest physiologically, given that it supports the idea that sympathetic control of blood pressure and blood flow is lateralized, at least with respect to the vestibulosympathetic reflexes studied.

It is generally assumed that sympathetic nerve activity is symmetrical: burst rates and burst amplitude distributions of MSNA have been shown to be similar on the two sides (59, 60); the same has been shown for SSNA (61). However, apart from our own observations (58), only one other study has demonstrated lateralization of sympathetic outflow. Diedrich et al. (62) found differential expression of MSNA on the left and right sides during sinusoidal neck suction, abolishing the normally right-sided dominance of carotid sinus baroreceptors on MSNA.

As noted above, the otoliths, rather than the semicircular canals, are believed to be primarily responsible for vestibulosympathetic reflexes (6, 10). And while GVS affects the firing of vestibular afferents originating in all parts of the vestibular apparatus (35, 36), recent evidence supports the idea that sGVS acts only *via* the otolith organs (38, 40). However, what we do not know is whether it is the utricular or saccular components of the otolith organs that are mediating the vestibulosympathetic reflexes. This requires a different means of vestibular afferent stimulation, one that can eliminate the semicircular canals and differentiate between the otolith organs.

## Linear Acceleration

Linear acceleration is a natural means of activating the vestibular apparatus. Yates et al. (63) had demonstrated increases in blood pressure and heart rate during linear acceleration (200 mG), and that these cardiovascular responses were absent in patients with bilateral loss of vestibular function. Jauregui-Renaud et al. (64) found similar results, control subjects exhibiting a sustained increase in heart rate and transient increase in breathing during linear acceleration (260 mG) that were absent in patients with vestibular dysfunction. These and other studies (63, 65–68) provide good evidence supporting the contribution of the otoliths to cardiovascular control. Direct recordings of MSNA during sinusoidal linear acceleration were first reported by Cui et al. (69), who found that MSNA decreased in subjects exposed to five cycles acceleration (100, 150, and 200 mG) in both the anteroposterior and medio-lateral directions (69, 70). However, all of these studies used fairly high accelerations (100–260 mG), which in addition to activating the vestibular organs will also activate extra-cranial receptors, such as those responsive to fluid shifts.

To circumvent this problem, we recently undertook a series of experiments that used low-amplitude (4 mG), low-frequency (0.08 Hz), sinusoidal linear acceleration of the body, seated on a motorized platform. By positioning the head vertically linear acceleration in the horizontal plane targets the utricular component of the vestibular apparatus (71, 72). These studies demonstrated a robust modulation of MSNA (32 ± 3 and 29 ± 3% for the *X* and *Y* axes), which was even higher for SSNA (97 ± 3 and 91 ± 5%, for the *X* and *Y* axes). Although there were no significant differences in amplitude of the modulation when delivered in the *X* or *Y* axes, the magnitude of modulation was markedly different between the two systems of sympathetic outflow. This can be simply due to the fact that the predominant influence on muscle vasoconstrictor drive is the arterial baroreceptors—cardiac modulation of MSNA is much greater than vestibular modulation of MSNA. Cardiac modulation is also higher than respiratory modulation of MSNA, which is of comparable amplitude to the respiratory and cardiac modulation of SSNA (73). In addition, while individual utricular afferents exhibit directional sensitivity, as a population there is no directional preference for evoking vestibulosympathetic reflexes. A noteworthy observation from these studies is that while the mean modulation indices produced by sinusoidal linear acceleration in the *X* and *Y* axes showed similar distributions across subjects, individual subjects could exhibit larger changes in sympathetic modulation in one axis than another. Indeed, it has been suggested that individual experiences may modulate the responses of vestibular hair cells responses and, hence, the magnitude of vestibulosympathetic responses (63).

In another series of experiments, subjects were supine with the neck aligned with the spine: sinusoidal linear acceleration, at the same amplitude and frequency, in the rostro-caudal (X) direction (longitudinal axis of the body) excites the saccular hair cells (although not exclusively). Cross-correlation analysis revealed modulation of MSNA (29% in the *X*-axis) that was no different to that produced by selective stimulation of the utricle (32% in the *X*-axis, 29% in the *Y*-axis), nor was it significantly different from that produced by acceleration of the supine body in the medio-lateral (*Y*) axis (32%), in which both the saccule and utricule are involved (72). This shows that both saccular and utricular organs contribute to the generation of vestibulosympathetic reflexes. This is also evident in the results from the composite sequences, reported by Grewal and colleagues, in which sinusoidal displacements of seated subjects were delivered in the *X* and *Y* axes (71).

**Figure 8:**
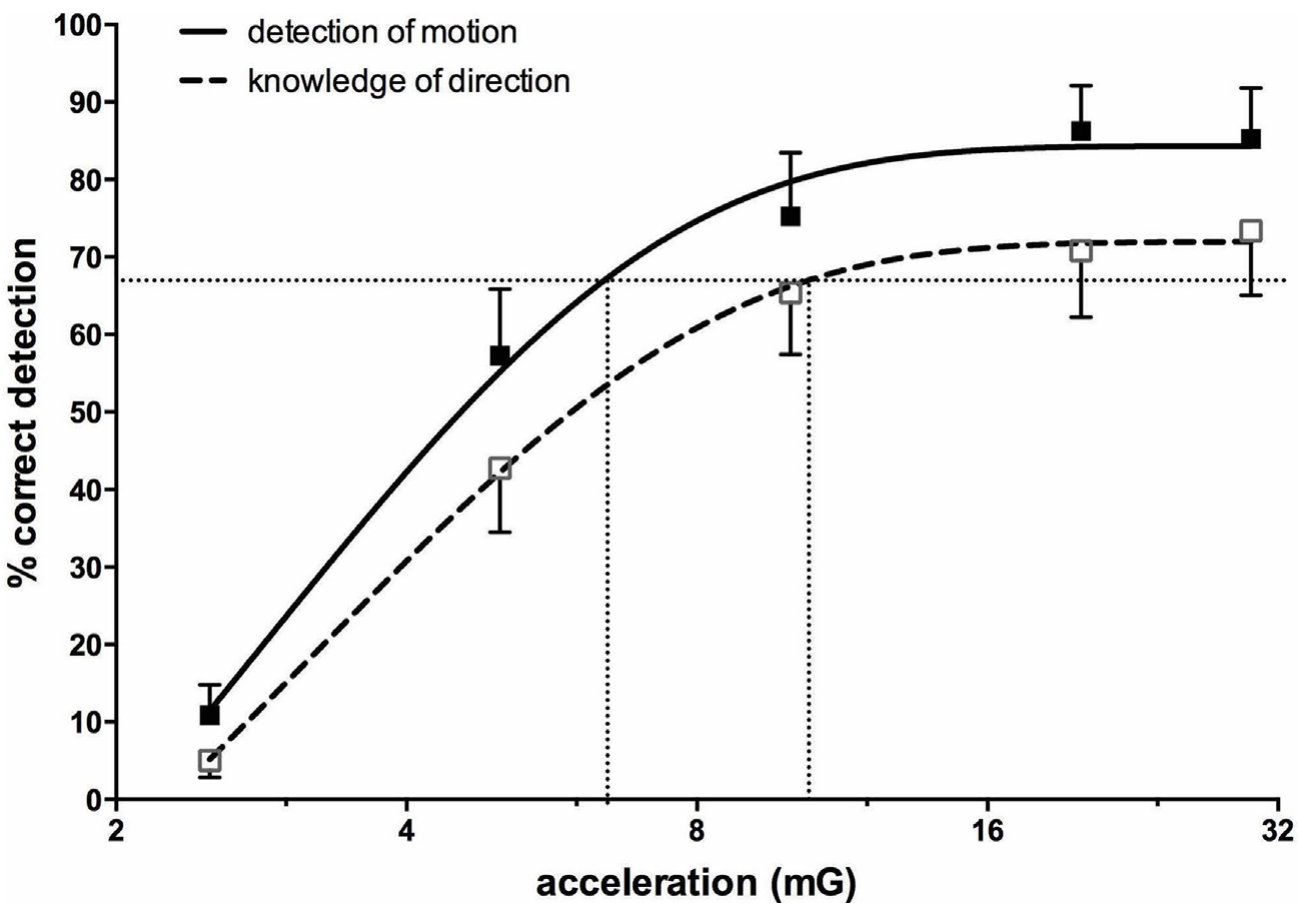
Percentage correct (mean ± SE) detection of motion, and correct detection of the direction of motion, for 16 subjects exposed to sinusoidal linear acceleration at a constant rate of 0.2 Hz but at accelerations ranging from 1.25 to 30 mG. Semi-logarithmic plot of data from 2.5 to 30 mG, with fitted sigmoidal curves shown superimposed. Reproduced with permission from Hammam et al. (74).

**Figure 9:**
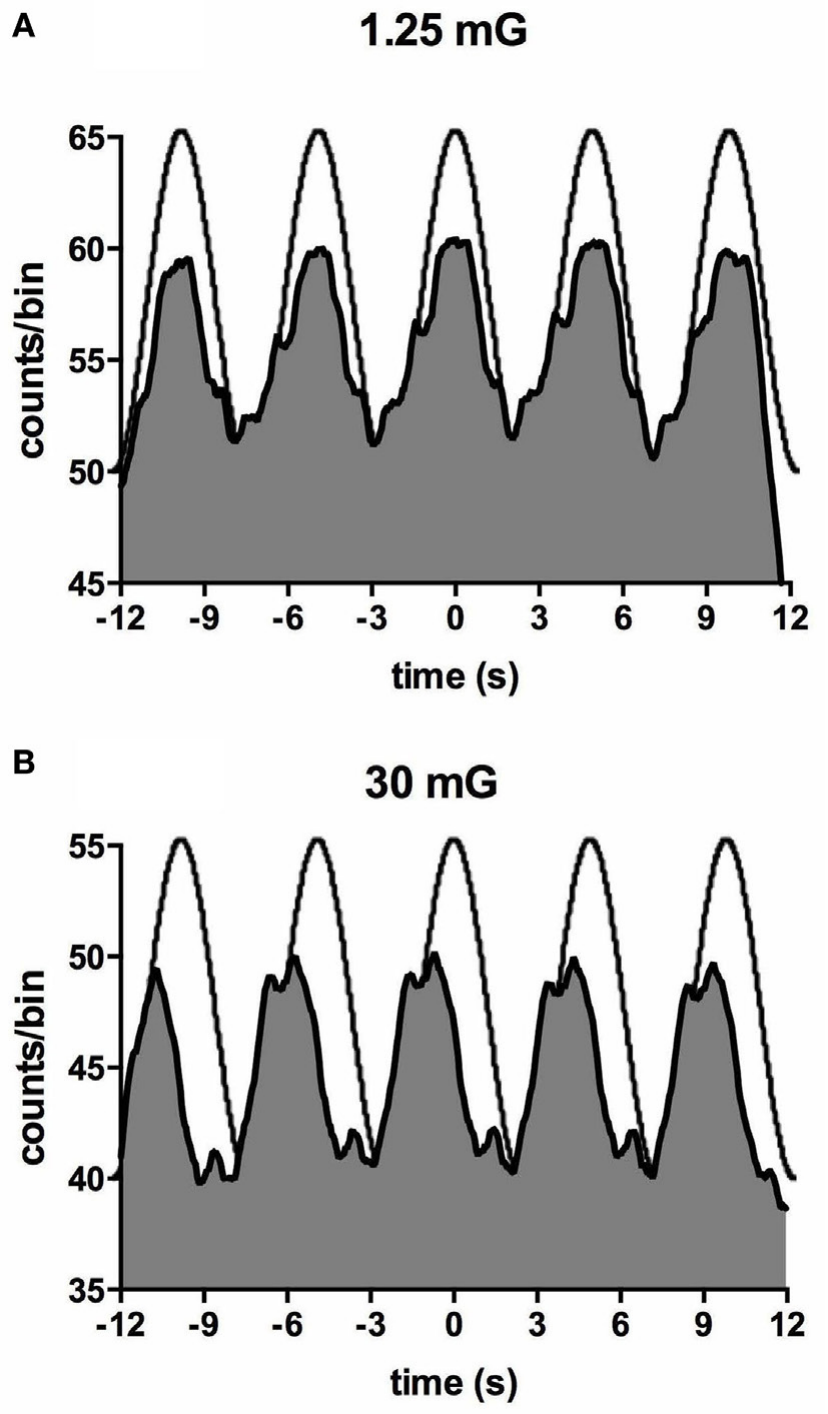
Cross-correlation histogram between muscle sympathetic nerve activity and acceleration in the anteroposterior direction for one subject exposed to accelerations of 1.25 mG **(A)** and 30 mG **(B)**. The histograms have been fitted with a smoothed polynomial. The superimposed sinusoid schematically represents the acceleration profile of the platform: motion in the forward direction is indicated by the positive phase of the sinusoid, which includes the period of acceleration before the peak and deceleration after the peak. Reproduced with permission from Hammam et al. (74).

Importantly, most subjects noted that they could not feel any motion, and if they did they could not tell in which direction they were moving (71, 72). To further quantify the capacity for subjects to perceive motion and accurately detect the direction of displacement during sinusoidal linear acceleration, we exposed participants to a range of acceleration amplitudes, extending from 1.25 to 30 mG at 0.2 Hz. As illustrated in Figure 8, the average threshold required to be able to detect the motion is 6.5 mG, while the acceleration required to accurately determine the direction of motion is 10.2 mG (74). Despite the fact that subjects could not perceive motion <6 mG, vestibular modulation of MSNA was apparent even at the lowest acceleration tested—1.25 mG (74). Modulation of MSNA at 1.25 mG and 30 mG is shown for one subject in Figure 9.

**Figure 10:**
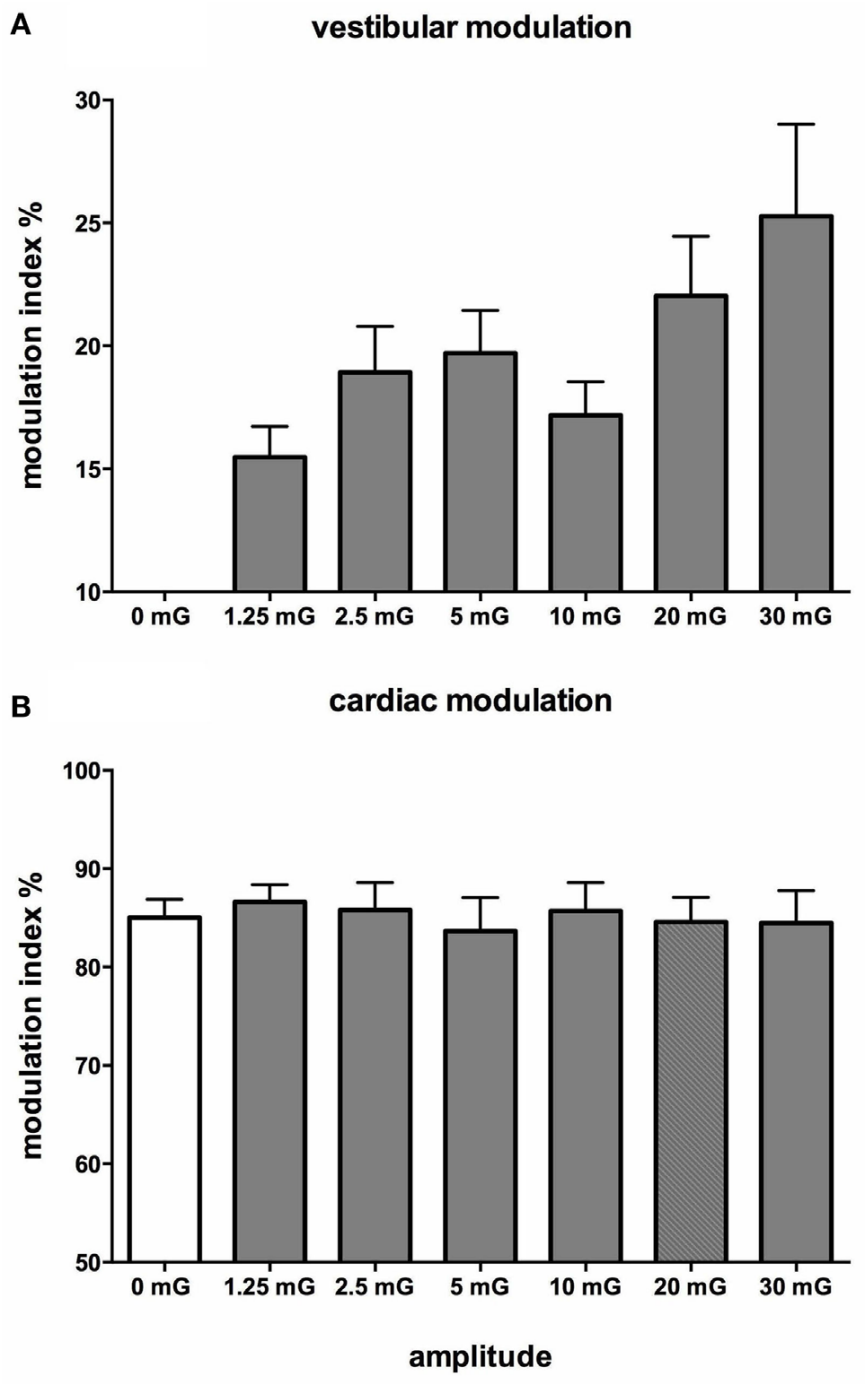
Mean vestibular **(A)** and cardiac **(B)** modulation indices of muscle sympathetic nerve activity as a function of acceleration amplitude; 0 mG = static condition (vestibular modulation = 0 in the absence of a sinusoidal vestibular input). Mean ± SE data from 13 subjects. Reproduced with permission from Hammam et al. (74).

Figure 10 shows mean data from all subjects: there was a positive slope of the magnitude of modulation as a function of acceleration amplitude. Based on studies in the monkey higher acceleration amplitudes would be expected to generate a greater vestibular input and thereby a greater modulation of sympathetic nerve activity Fernández and Goldberg (75). These authors used accelerations up to 5 G—*two orders of magnitude higher than that discussed here*. The outcome of this study highlights that the vestibular afferents certainly do respond to the higher accelerations (30 mG), but more importantly, they also respond during acceleration of the lowest magnitude (1.25 mG). That we observed robust modulation of MSNA during very low amplitude, sub-perceptual sinusoidal motion, indicates that the modulation of MSNA was not due to any conscious awareness or arousal-related component and purely reflects the expression of a vestibulosympathetic reflex. Indeed, detection of motion did not occur until accelerations of ~6.5 mG, with knowledge of the direction of movement not being apparent until ~10 mG. However, while these reflexes are robust, it is worth pointing out that they are certainly smaller than the baroreceptor-mediated reflexes: as seen in Figure 10, cardiac modulation was much higher than the vestibular modulation and was not affected by the amplitude of acceleration. Overall, this highlights the exquisitely rapid detection of acceleration by the vestibular hair cells; however, this seems to only be of importance at larger postural changes when immediate blood pooling is compromised, until the relatively slower unmyelinated baroreceptor fibers unload (76–78).

## Contributions of Neck Afferents

**Figure 11:**
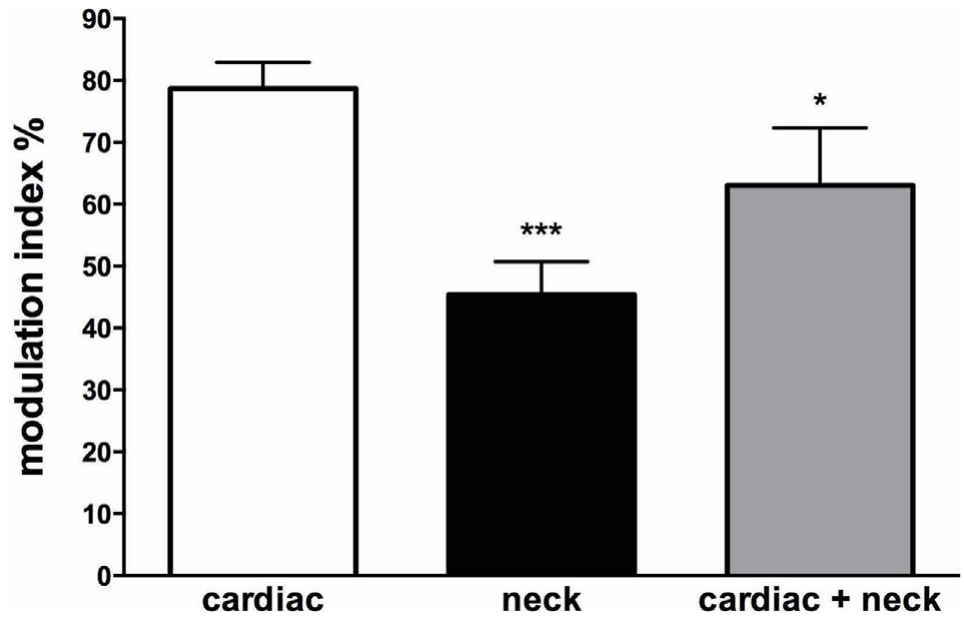
Mean modulation indices (see Methods) calculated from the cross-correlation histograms between muscle sympathetic nerve activity (MSNA) and ECG at rest (*cardiac*), MSNA and neck angle during sinusoidal neck displacement (*neck*) and between MSNA and ECG during sinusoidal neck displacement (*cardiac + neck*). Neck modulation of MSNA was significantly lower than cardiac modulation at rest and cardiac modulation was significantly lower during neck stimulation. *P < 0.05; ***P < 0.001. Reproduced with permission from Bolton et al. (79).

Interestingly, there are some circumstances when the head moves (and, hence, the vestibular system is engaged) but the body does not move—such as lifting the head while lying supine. In these circumstances, there is no need to increase vasomotor tone to the lower limbs. So the question arises, how does the brain know when it is appropriate to modulate vasomotor tone to the lower limbs of the habitually upright human? We recently examined the influence of neck afferents on MSNA by employing sinusoidal displacement of the body about the neck—and reported two new findings. First, neck proprioceptors can modulate MSNA in the lower limbs of awake humans and, second, the cardiac modulation of MSNA is reduced in the presence of neck modulation of MSNA [Figure 11 (79)]. While a previous study had examined the influence of neck afferent input on MSNA, they found that neck afferents did not modulate lower limb MSNA (80). However, a significant difference is that the Ray and Hume studies analyzed the MSNA of subjects during *static* flexion or extension of the head and neck while subjects were in the lateral decubitus or supine position, respectively. By contrast, Bolton et al. (79) recorded MSNA during *dynamic* stretching of the neck. That the stimulus may need to be dynamic in order to have an effect on MSNA has been observed with respect to vestibular modulation of MSNA in humans. *Dynamic* (sinusoidal or trains of pulses) GVS modulates MSNA in the human (46, 81) but *static* (1 s step) stimuli fail to do so (41). This makes teleological sense since *dynamic* changes in posture (body position) are more likely to require modulation of vascular tone than during static states. However, further research is required to determine if the magnitude of neck modulation of MSNA in postures that are likely to induce an orthostatic challenge is sufficient to increase vasomotor tone in the lower limbs and thereby reduce the likelihood of orthostatic hypotension.

Moreover, cardiac modulation of MSNA was reduced in the presence of *dynamic* neck stretch. Animal studies suggest that the vestibular nuclei and regions of the brainstem are involved in integrating information from both somatic and visceral sources and higher centers in order to regulate blood pressure in different body positions and contexts (82–84). The cross-correlation analysis in Bolton et al. (79) showed cyclical modulation of MSNA in all subjects during neck muscle stretching induced by sinusoidal body displacement about the fixed head. This most likely involved the pathways that mediate vestibulosympathetic reflexes (85). Experimental evidence from animal studies have shown that neck muscle spindle afferents, which are exquisitely sensitive length sensors (86), project to the medial and descending vestibular nuclei and can modulate vestibular neuronal activity (87, 88). Electrical stimulation of nerves innervating muscles of the neck at currents just sufficient to stimulate muscle spindle (and Golgi tendon organ) afferents has been shown to change activity in respiratory and sympathetic nerves in the cat (89). Moreover, it has been shown that the presence of intact neck afferents inhibits the influence of the vestibular system on sympathetic outflow during natural stimulation of the labyrinths (89).

It should be pointed out, however, that sinusoidal displacement of the body about the neck might change transmural pressures at the carotid sinus and, hence, change the input from the arterial baroreceptors. Not withstanding the fact that the phasic cardiac modulation of MSNA was much greater than that produced by sinusoidal neck movements in the study by Bolton et al. (79), or by electrical or physiological activation of the otolithic organs in previous studies, Shortt and Ray (15) discounted changes in carotid baroreceptor input in their experiments by arguing that there were no changes in blood pressure and, because there were no changes in thoracic volume, discounted any contribution from the low-pressure baroreceptors.

## Clinical Considerations

The physiological adjustments to a hydrostatic challenge imposed during postural changes is predominantly a function of the baroreflex mechanism, with afferent contributions from the vestibular system (defining head acceleration) and the neck afferents (defining the position of the body in space). Clinical studies are limited, and while a lot of work has been conducted in quadrupedal mammals, its translation to bipedal humans is somewhat limited.

Nevertheless, we know that acute vestibular lesions produce distressing symptoms of nausea, vomiting, tachycardia, and palpitations—all of which are autonomic markers (29). A study that examined patients with acute vertigo due to unilateral vestibular neuritis (48 h from onset) showed a depression in sympathetic reactivity to orthostatic challenges—a dysfunction that resolved in 2 weeks (65). Similarly, patients with bilateral loss of vestibular function, when exposed to linear motorized accelerations, exhibit an inadequacy in cardiovascular control when compared with healthy controls (64). Moreover, on their return from space, astronauts face the challenge of deconditioned gravitational accelerometers—the otolith organs—resulting in orthostatic intolerance, though resolving 10 days after return (31). The short-lasting dysfunction is echoed and well described in the animal literature, but little is known about the replacement or recovery mechanism following vestibular damage in humans (3).

However, aging-related orthostatic hypotension is a chronic condition and is commonly found in the elderly (90). It is derived from multiple etiologies, including sympatholytic drugs associated with comorbidities, but it has been documented that vestibulosympathetic reflexes depresses with increase in aging (18, 19), suggesting a co-contribution to the debilitating effects of orthostatic intolerance in the elderly. It is, however, an understudied area and requires further investigations to establish a better understanding of the relationship of vestibulospinal reflexes and aging-related orthostatic intolerance.

## Conclusion

It is now abundantly clear that the vestibular system can modulate sympathetic outflow to both muscle and skin. While our use of low-frequency sGVS has shown that sympathetic outflow can be strongly entrained to vestibular inputs, it is our use of low-frequency sinusoidal linear acceleration that has revealed that both the utricular and saccular components of the vestibular apparatus are responsible for the generation of vestibulosympathetic reflexes. Given that the otolithic organs encode both static position and linear acceleration of the head in space, these findings emphasize that the vestibular apparatus contributes to the control not just of motoneurones involved in posture and locomotion but also sympathetic neurones involved in the control of blood pressure. However, why it should also influence sympathetic outflow to the skin is more difficult to understand. It may well be that vestibular modulation of SSNA has no physiological significance, and may simply reflect coupling of control mechanisms between thermoregulation and blood pressure regulation. Indeed, given that vestibular modulation of muscle sympathetic outflow clearly does play a role in the regulation of blood pressure, and that the distribution of cardiac output to skeletal muscle and skin needs to be controlled with the opposing regulatory demands of blood pressure and body temperature, it makes physiological sense for there to be some common mechanisms for controlling blood flow in muscle and skin when these two demands compete, such as in exercise.

## Author Contributions

EH drafted the manuscript. VM contributed to the writing. Both authors approved the final manuscript.

## Conflict of Interest Statement

The authors declare that the research was conducted in the absence of any commercial or financial relationships that could be construed as a potential conflict of interest.
